# Critical assessment of coalescent simulators in modeling recombination hotspots in genomic sequences

**DOI:** 10.1186/1471-2105-15-3

**Published:** 2014-01-03

**Authors:** Tao Yang, Hong-Wen Deng, Tianhua Niu

**Affiliations:** 1 Center for Bioinformatics and Genomics, Department of Biostatistics and Bioinformatics, Tulane University School of Public Health and Tropical Medicine, 1440 Canal Street, Suite 2001, New Orleans, LA 70112, USA

**Keywords:** Coalescent, Population genetics, Linkage disequilibrium, Recombination, Single nucleotide polymorphism

## Abstract

**Background:**

Coalescent simulation is pivotal for understanding population evolutionary models and demographic histories, as well as for developing novel analytical methods for genetic association studies for DNA sequence data. A plethora of coalescent simulators are developed, but selecting the most appropriate program remains challenging.

**Results:**

We extensively compared performances of five widely used coalescent simulators – Hudson’s ms, msHOT, MaCS, Simcoal2, and fastsimcoal, to provide a practical guide considering three crucial factors, 1) speed, 2) scalability and 3) recombination hotspot position and intensity accuracy. Although ms represents a popular standard coalescent simulator, it lacks the ability to simulate sequences with recombination hotspots. An extended program msHOT has compensated for the deficiency of ms by incorporating recombination hotspots and gene conversion events at arbitrarily chosen locations and intensities, but remains limited in simulating long stretches of DNA sequences. Simcoal2, based on a discrete generation-by-generation approach, could simulate more complex demographic scenarios, but runs comparatively slow. MaCS and fastsimcoal, both built on fast, modified sequential Markov coalescent algorithms to approximate standard coalescent, are much more efficient whilst keeping salient features of msHOT and Simcoal2, respectively. Our simulations demonstrate that they are more advantageous over other programs for a spectrum of evolutionary models. To validate recombination hotspots, LDhat 2.2 rhomap package, sequenceLDhot and Haploview were compared for hotspot detection, and sequenceLDhot exhibited the best performance based on both real and simulated data.

**Conclusions:**

While ms remains an excellent choice for general coalescent simulations of DNA sequences, MaCS and fastsimcoal are much more scalable and flexible in simulating a variety of demographic events under different recombination hotspot models. Furthermore, sequenceLDhot appears to give the most optimal performance in detecting and validating cross-over hotspots.

## Background

Coalescent simulation is a very useful tool in population genetics with a rich variety of applications, particularly to evaluate and compare performances of various statistical methods in rare variant analysis [1-3], to estimate parameters for different population histories [4,5] and to infer phylogenetic trees [6]. In simulating DNA sequence data for studying human complex diseases, recombination hotspots, defined as genomic intervals with local recombination rates increased relative to that of the surrounding DNA region, need to be taken into account given their ubiquity in the human genome [7]. Further, population geneticists are interested in examining haplotype block patterns delineated by recombination hotspots along chromosomes in fine-mapping locations of disease loci. Over the past decade, a plethora of coalescent simulators have been developed, e.g. SelSim [8], CoaSim [9], FastCoal [10], Mlcoalsim [11], and RECOAL [12], to name a few. Of them, five most representative and widely used coalescent simulators, Hudson’s ms [13], msHOT [14], Markovian Coalescent Simulator (MaCS) [15], Simcoal2 [16], and fastsimcoal [17].

**Figure 1:**
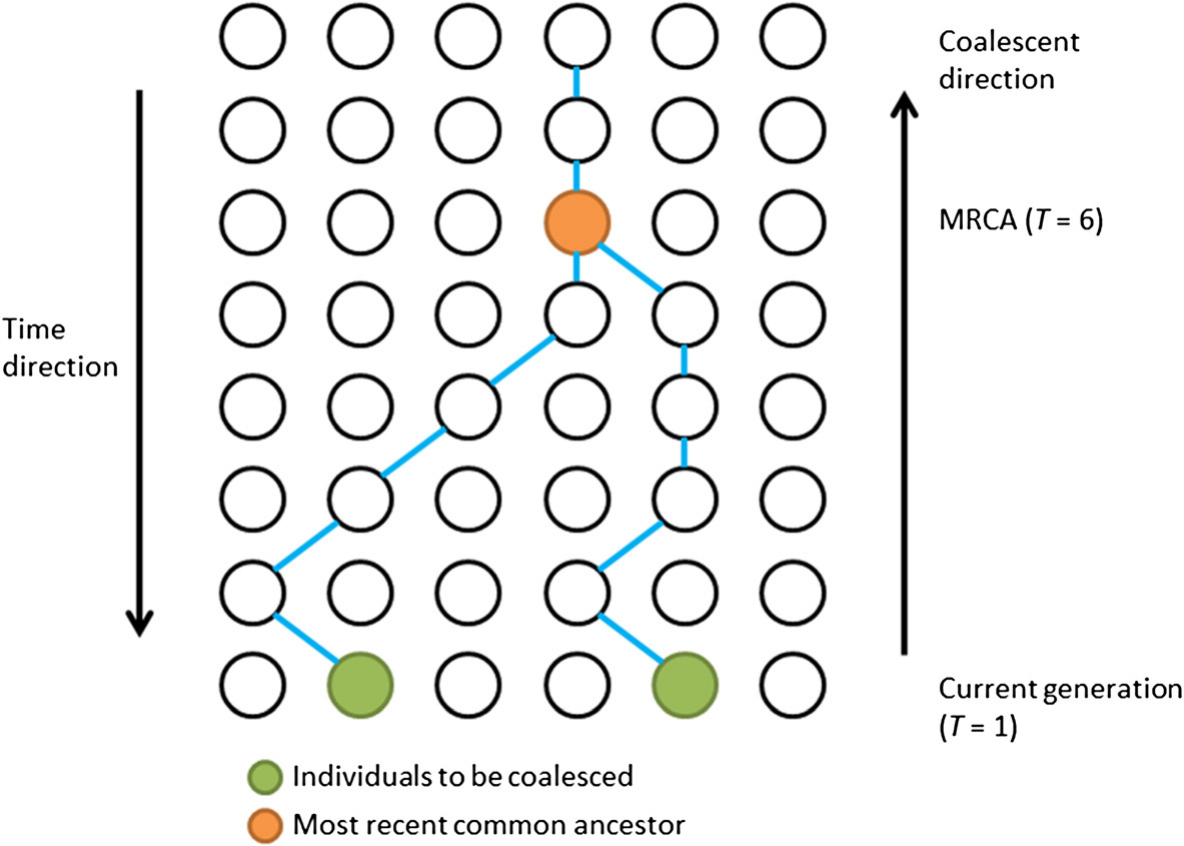
**Kingman’s coalescent process. It starts from the current generation (bottom) tracing backward in time to the most recent common ancestral (MRCA, orange solid circle).** Two individuals (green solid circles) coalesced at the sixth generation backward time. The distributions of allele frequencies in successive generations follow a Wright-Fisher model.

**Figure 2:**
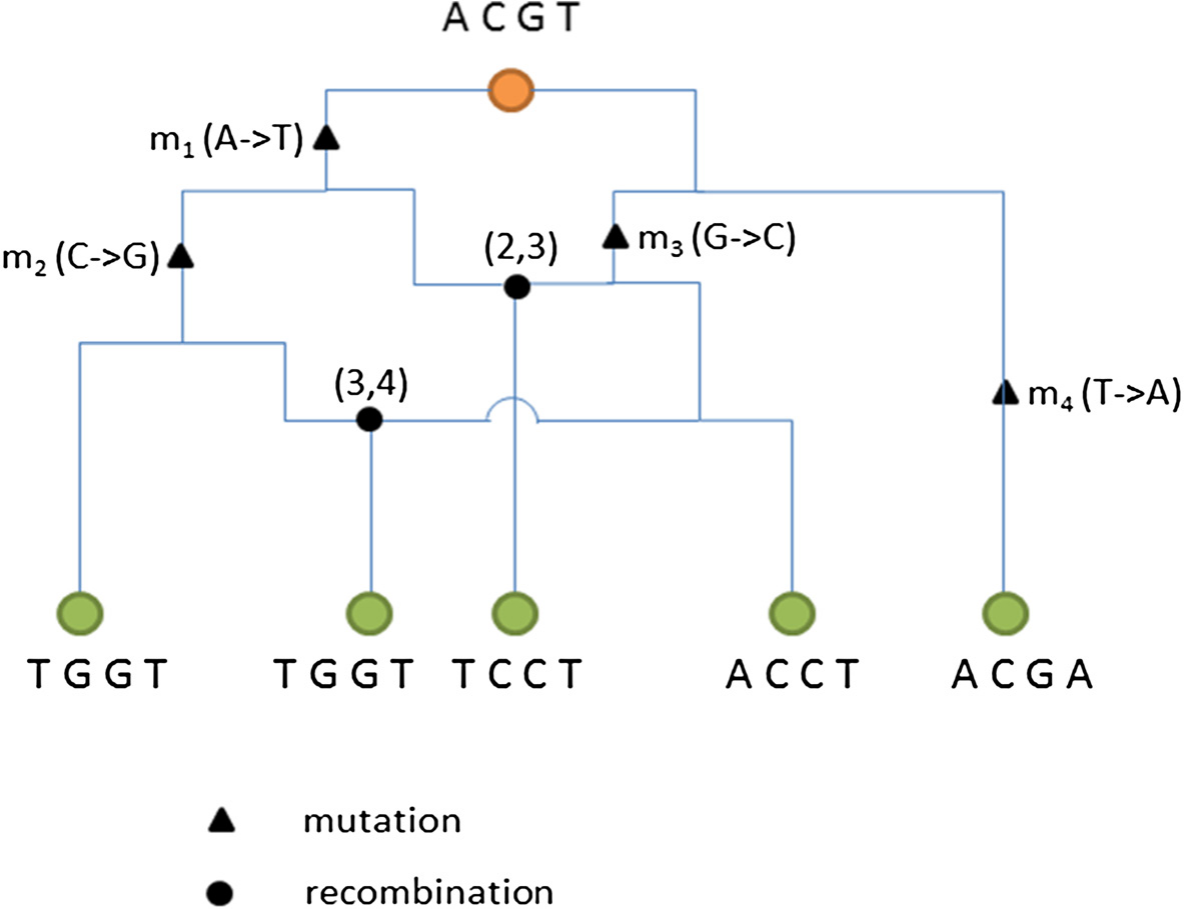
**A simple ancestral recombination graph for illustrative purpose. The ancestral sequence is “ACGT” (top).** After four mutations (denoted by *m*_*1*_, *m*_*2*_, *m*_*3*_, and *m*_*4*_, respectively) and two recombination events [denoted by (2, 3) and (3, 4), respectively], it has evolved into five (four distinct) present-day sequences, i.e., “TGGT”, “TGGT” (i.e., same as the sequence to the left), “TCCT”, “ACGT”, and “ACGA”. Notation *m*_*1*_ indicates an A→T mutation on locus 1. Notation (2, 3) denotes a recombination between alleles at loci 2 and 3.

Coalescent process, based on the neutral Wright-Fisher Model, was first introduced by Kingman [18,19] (Figure 1). Simulating the entire ancestral recombination graph (ARG) [13,20] encompassing all coalescent and recombination events in the past for a sample of DNA sequences imposes a considerable computational burden. In 2002, Hudson [13] developed ms, a Monte Carlo program based on standard coalescent that assumes an infinite-sites model for mutational events, which allows for recombination and gene conversion events, as well as symmetric migrations of subpopulations. While the standard coalescent starts at the present time and simulates backward in time, Wiuf and Hein (1999) are the first to introduce a sequential interpretation of the coalescent with recombination [21], such that genealogies could be simulated by moving along a DNA sequence. Based on the Wiuf and Hein algorithm, McVean and Cardin in 2005 developed a sequential Markov coalescent (SMC) method [22]. The SMC starts with a coalescent tree at the left-hand end of the sequence and progressively modifies the tree with recombination events as it moves to the right, and has been shown to produce patterns of polymorphisms and linkage disequilibrium (LD) extremely similar to those generated under a classical ARG model [10,15,23]. SMC’ , an improved algorithm based on the SMC method, was developed by Marjoram and Wall in 2006 [10], such that a lineage separated by a recombination event is allowed to also attach to its old path (Figure 2). While retaining the speed and memory efficiency of SMC, SMC’ appears significantly more accurate when benchmarked by the exact coalescent process. MaCS, also a variant of the Wiuf and Hein algorithm, generates simulated data that are virtually identical to data simulated under the standard coalescent, but in much less time and using much less memory. MaCS is considered as a “generalized” SMC, which could provide a better approximation to results of standard coalescent than SMC, by tuning a user-specified “history” parameter *h* denoting a sequence length [15]. Taken together, both SMC’ and MaCS give closer approximations to standard coalescent than SMC.

Although a set of DNA sequences with a pre-determined number of recombination hotspots at user-defined positions could be generated by msHOT, MaCS, Simcoal2, and fastsimcoal, there appears to be a lack of rigorous validation of hotspot detection software tools in the current literature. Popular computer programs for discovering recombination hotspots include LDMAP [24], LDhot [7,25], LDhat 2.2 rhomap package [26], Hotspotter [27], and sequenceLDhot [28]. The widely used Haploview program [29,30] is also an appealing tool to visually localize recombination hotspots given the delineated LD block structure. Based on popularity, LDhat 2.2 rhomap, sequenceLDhot, and Haploview programs were chosen for detecting recombination hotspots in both real data and simulated data generated by the coalescent process. Because sequenceLDhot gave the best performance in inferring locations of cross-over hotspots, recombination hotspots simulated by msHOT, MaCS, and fastsimcoal were subject to validation by sequenceLDhot.

## Methods

### Coalescent process

Coalescent process was initially derived as an approximation of the neutral Wright-Fisher model. This approximation works well when sample sizes are small relative to the population size. Mutations are assumed to be Poisson distributed along each branch given the mutation rate and branch length. Normally, an infinite-sites model [31] is assumed, which means no recurrent mutations occur. Each recombination event breaks the sequence into several segments, and each segment is modeled by a genealogy tree. Simulation of recombination hotspots is realized by changing the rates where these recombination events occur. The process that includes both mutation and recombination events is illustrated by ARGs (Figure 2). The SMC is an approximating algorithm for simulating a series of trees that differ from each other by a single recombination event, starting from the left end and moving to the right end of the DNA sequence.

### Wright-Fisher model

Consider a population of constant size consisting of *N* diploid individuals, which means there are *2N* copies for a given gene. Generations are assumed to be non-overlapping and denoted by *T* = 1, 2, …. Each individual in the next generation receives two copies of the gene (one from each parent) and for each respective parental copy, the gene is selected randomly and with replacement from the two copies of the gene present among the parents. At time *T* = 1, without loss of generality, assume *i* (e.g., *i* =2) of these gene copies are of type ***A***. Let *Y*_*T*_ denote the random variable for the number of type ***A*** gene at the time of *T,* then *Y*_*T*_*|Y*_*T-1*_*= y*_*T-1*_ ~ *Bin* (*2N, y*_*T-1*_*/2N*), such that

$$P\left(Y_{T}=y_{T}|Y_{T-1}=y_{T-1}\right)=\left(\begin{array}{c} 2N\\ y_{T} \end{array}\right){\left(\frac{y_{T-1}}{2N}\right)}^{y_{T}}{\left(1-\frac{y_{T-1}}{2N}\right)}^{2N-y_{T}},$$

where *y*_*T*_ denotes the realized value of the random variable *Y*_*T*_.

Based on this model, the trajectory of the coalescent process (Figure 1) tracing from the current generation backwards in time to the generation where ***A*** is coalesced could be modeled. The current generation could then be simulated through this trajectory given a random seed.

### ARG

Figure 2 illustrates a simplest ARG [32]. Take the third 4-letter sequence “TCCT” as an example. The common ancestral sequence evolves into two branches. For each branch, mutations have taken place on alleles at loci 1 and 3, respectively, giving rise to “TCGT” and “ACCT”. Next, a recombination event arises between these two sequences on (2, 3), and “TCCT” is produced. In the standard coalescent, a full ARG delineating all past coalescent and recombination events is constructed, with simulated samples corresponding to the edges of the graph.

### SMC approach and its two variants (SMC’ and MaCS)

**Figure 3:**
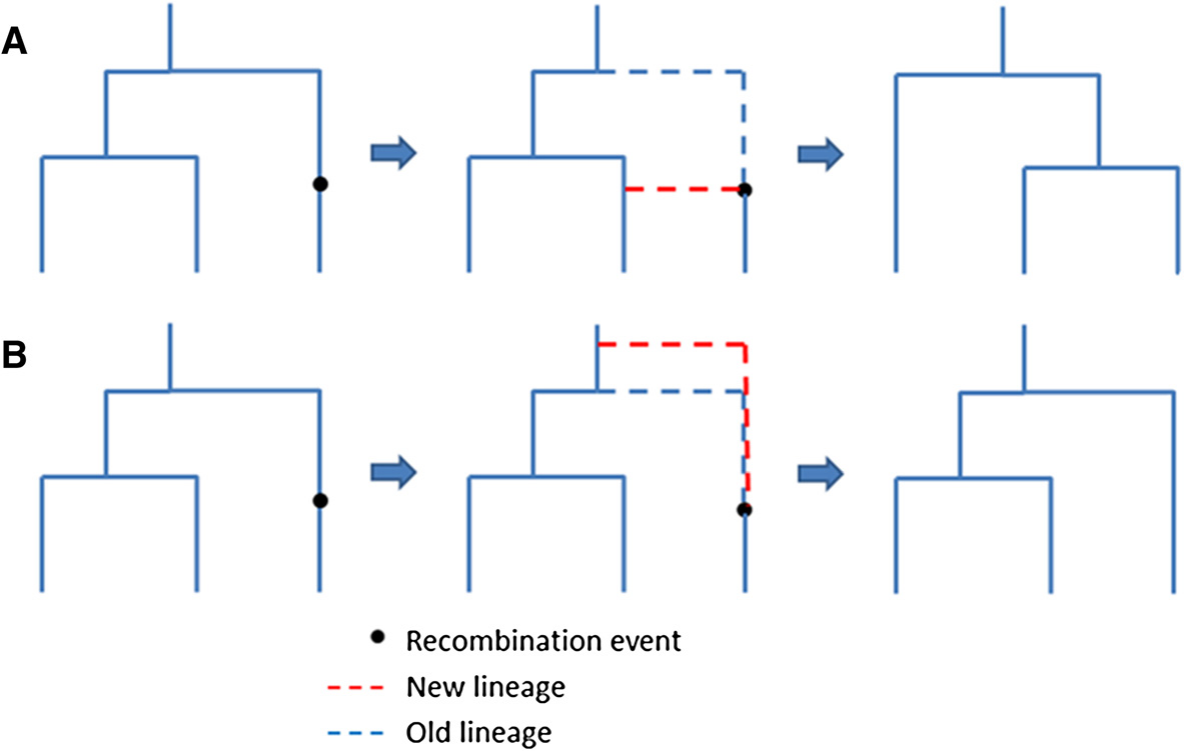
**Illustrations of Sequential Markov Coalescent (SMC) and the variant SMC’ algorithms. (A)** Re-attachment process of SMC, such that a cut lineage was re-attached to the remaining tree. **(B)** Re-attachment process of a variant of SMC named SMC’ , such that a re-attaching to the old path was allowed.

In contrast to standard coalescent which starts at the present time and is simulated backwards in time, the SMC approach [22] builds a coalescent tree by moving along the DNA sequence starting from the left end, and progressively updates the tree by incorporating recombination event(s) until the right end of the tree is reached. The region flanked by two neighboring recombination events is modeled by a local tree. Recombination is assumed to follow an exponential distribution along the sequence. A recombination event occurs randomly along the current tree, and the detached recombining lineage is free to coalesce with the other remaining lineages, leading to a new tree with a potentially different topology and most recent common ancestor (MRCA) (Figure 3A). SMC’ [10], a variant of SMC, allows the detached lineage cut by recombination event to coalesce with the exact same branch where it is detached from (Figure 3B), and results in a closer approximation to exact standard coalescent. MaCS algorithm [15], also a modified SMC algorithm, further allows for coalescence with trees one recombination away on the left hand side, thus achieving a higher accuracy than SMC in approximating the standard coalescent. Fastsimcoal [17], a completely rewritten continuous-time implementation of Simcoal2 [16], implements a fast SMC’ that allows for multiple recombination events between markers at fixed recombination distances on the sequence.

### Detection of recombination hotspots

The LDhat 2.2 rhomap package [26], sequenceLDhot [28], and Haploview program [29,30] were applied respectively to detect recombination hotspots in the simulated sequences. LDhat is a popular software for estimation of recombination rates, which was developed based on Bayesian reversible-jump Markov Chain Monte Carlo (MCMC) algorithm, and rhomap is a new method incorporated into LDhat 2.2 that specializes in fitting cross-over hotspot model. Another widely used computer program in detecting recombination hotspots— sequenceLDhot, uses an approximate marginal likelihood method of [33] to estimate a likelihood ratio (LR) statistic to unveil a cross-over hotspot. Further, Haploview was implemented to obtain a visualization of the LD block structure that could reflect varying recombination rates along a contiguous DNA sequence.

### Real data set

The 216-kb human leukocyte antigen (HLA) class II region is a well-studied region where recombination hotspots have been identified with sperm typing technology [34-36]. The original data set analyzed in the current study (http://www.le.ac.uk/ge/ajj/HLA/Genotype.html) contains genotype data for 50 unrelated UK Caucasians for 296 markers [i.e., 264 single nucleotide polymorphisms (SNPs) and 22 1–11-bp insertion/deletion polymorphisms] [35]. A subset of 263 SNPs without missing data were selected for recombination hotspot detection. Multi-locus haplotypes are required as an input for hotspot detection programs such as sequenceLDhot, and they provide crucial phase information that is important for understanding haplotype structure [37,38]. Therefore, haplotypes across the 263 SNPs were reconstructed by PHASE v2.1 program, a haplotype inference method based on the (i) coalescent theory using a variant of canonical Gibbs sampling [33,39], (ii) an LD decay model [40], and (iii) the partition-ligation algorithm [41]. In total, 100 haplotypes for the 216-kb region were statistically inferred.

### Simulation data sets

In order to assess the running efficiency with or without recombination hotspots, we simulated a set of DNA sequences (i.e., haplotypes) according to 0-, 2-, and 5-hotspot models using these five simulators respectively. All three hotspot models were simulated based on sequences with lengths 1- and 5-Mb, respectively. For each hotspot model and each sequence length, we simulated sample sizes (i.e., number of sequences) of 100, 500, 1,000, and 10,000, respectively. We implemented a symmetric two-island model with a total effective population size of 10,000. The recombination rate and mutation rate were both assumed to be 1.0 × 10^-8^ per site. Fifty replicates were done and the running times were recorded. All simulations were run on the platform — Linux OS, 2.0-GHz CPU, 1TB-RAM.

To validate the recombination hotspot position and intensity accuracy, we simulated the data sets according to 2- and 5-hotspot models along a 200-kb long DNA sequence. The recombination hotspots’ intensities were set to be 100 times higher compared to the background recombination rates.

## Results

### Features comparison

The features of the five simulators (i.e., ms, msHOT, MaCS, Simcoal2, and fastsimcoal) are shown in Table 1. Except for ms, all the other four simulators are able to simulate recombination hotspots with arbitrarily chosen recombination rates. Simcoal2 and fastsimcoal cannot simulate gene conversion events, whereas ms, msHOT, and MaCS could. MaCS, Simcoal2 and fastsimcoal allow stochastic uncertainty in sampling of segregating sites to be incorporated as a factor. For simulating rare mutation events, the desired allele frequencies could be preset for MaCS, Simcoal2 and fastsimcoal, but not for ms and msHOT. One of the advantageous features is that these coalescent simulators are able to simulate according to presumed migration patterns (e.g., between subpopulations residing in islands A and B), although the population structures vary to some degree — ms, msHOT and MaCS require these subpopulations to be symmetric across all islands (i.e., islands A and B must have equal sample sizes, but A→B and B→A migration rates could differ), while both Simcoal2 and fastsimcoal allow for arbitrarily chosen population structures (i.e., these programs allow for asymmetry). This feature makes the coalescent simulation a good choice for simulating admixture populations. Both Simcoal2 and fastsimcoal could simulate a mixture of different data types including DNA sequences, SNPs, microsatellites, which make them good tools for studying a variety of chromosome structures. In particular, the coalescent simulation strategy of fastsimcoal2 adopts a so-called “Approximate Bayesian Computation (ABC)” [42] to estimate the demographic parameters and the mutation and recombination rates, which has a unique feature that allows for sampling simulation parameters from predefined prior distributions, thus facilitating simulations for estimating the parameters. Because Simcoal2 implements a discrete time generation-by-generation simulation, it allows for multiple coalescent events per generation. However, the generation-by-generation simulation is very time-consuming, which significantly lowers its computational speed (see below).

**Table 1:** Feature comparisons of five widely used coalescent simulators

**Category**	**ms**	**msHOT**	**MaCS**	**Simcoal2**	**Fastsimcoal**
Hotspot	No	Yes	Yes	Yes	Yes
Gene Conversion	Yes	Yes	Yes	No	No
Ascertainment	No	No	Yes	Yes	Yes
Algorithm	SC^†^	SC	SMC’^#^	Gen-By-Gen*	SMC’
Admixture	Yes	Yes	Yes	Yes	Yes
Multiple event/Gen	No	No	No	Yes	No
Migration	Yes	Yes	Yes	Yes	Yes
Population structure	Symmetric	Symmetric	Symmetric	Arbitrary	Arbitrary
Different data types	No	No	No	Yes	Yes
Arbitrary pattern of recombination	No	Yes	Yes	Yes	Yes
Computation speed	Moderate	Moderate	Fast	Slow	Fast
Sampling simulation parameters	No	No	No	No	Yes
Publication Year	2002	2007	2009	2004	2011
# of Citations**	1,300	52	82	185	16

^†^Standard coalescent.

^#^Modified version of Sequential Markov Coalescent.

*Discrete time generation-by-generation simulation.

**Google Scholar (as of December 23, 2013).

### Running efficiency

For each design of simulation, we generated 50 replicates according to our simulation settings (see simulation data sets in Methods section) and the average execution times are presented in Tables 2 and 3. For simulations of a 1-Mb sequence (Table 2), all the simulations could be performed within 6 minutes. When sample size was increased to 10,000, both ms and msHOT gave a segmentation fault error on our platform (Linux OS, 2.0-GHz CPU, 1TB-RAM) when 0-hotspot model was simulated. For simulations of a 5-Mb sequence (Table 3), both ms and msHOT gave segmentation fault errors when the sample sizes exceeded 500. This indicates the limitations of ms and msHOT in simulating long sequences and for large sample sizes. It is interesting that when recombination hotspots are simulated, msHOT is able to simulate long DNA sequences for large sample sizes. This is because msHOT is exactly the same as ms when simulating a 0-hotspot model. When simulating 2- and 5-hotspot models, msHOT and Simcoal2 performed much slower than MaCS and fastsimcoal because of algorithmic differences. Further, when the number of hotspots got larger, msHOT performed slower than Simcoal2. To simulate 1,000 DNA sequences (i.e., haplotypes), it could take three and a half hours for msHOT, and one and a half hours for Simcoal2, respectively. In contrast, MaCS and fastsimcoal were much faster – simulating 1,000 of 5-Mb sequences under the 5-hotspot model took only 90 seconds for MaCS and 40 seconds for fastsimcoal, respectively. For all simulation settings, fastsimcoal always performed a little faster than MaCS (Tables 2 and 3). It is worthy to mention that increasing the number of hotspots did not affect the execution times for MaCS, Simcoal2, and fastsimcoal, but did so for msHOT.

**Table 2:** Average (50 replicates) execution time (standard deviation) of simulating 1-Mb sequence data with a pre-specified number of recombination hotspots (mm:ss)*

**Number of hotspots**	***N***	**ms**	**msHOT**	**MaCS**	Simcoal2^*^	**Fastsimcoal**
0	100	0:01 (2×10^-3^)	0:02 (0:01)	0:01 (0:00)	1:51 (0:03)	<0:01 (4×10^-4^)
	500	0:01 (2×10^-3^)	0:04 (0:01)	0:06 (0:01)	2:19 (0:04)	0:03 (3×10^-3^)
	1,000	0:02 (5×10^-3^)	0:06 (0:01)	0:23 (0:02)	2:34 (0:08)	0:08 (0:01)
	10,000	---	---	5:42 (0:13)	3:51 (0:07)	2:18 (0:03)
2	100	---	1:28 (0:02)	0:03 (0:01)	1:51 (0:06)	<0:01 (3×10^-4^)
	500	---	1:43 (0:05)	0:10 (0:01)	2:45 (0:08)	0:03 (0:01)
	1,000	---	1:51 (0:05)	0:25 (0:03)	2:50 (0:09)	0:08 (0:01)
	10,000	---	2:39 (0:07)	5:43 (0:10)	4:27 (0:11)	2:22 (0:03)
5	100	---	2:48 (0:05)	0:02 (0:01)	2:30 (0:06)	<0:01 (9×10^-4^)
	500	---	3:14 (0:07)	0:10 (0:02)	2:41 (0:05)	0:03 (5×10^-3^)
	1,000	---	3:33 (0:06)	0:23 (0:02)	3:24 (0:07)	0:09 (0:02)
	10,000	---	4:27 (0:11)	5:36 (0:15)	4:10 (0:09)	2:32 (0:05)

* “*N*” denotes sample size (i.e., the number of DNA sequences) for each replicate. “---” denotes a segmentation fault (e.g., ms could not simulate sequence data with recombination hotspots).

**Table 3:** Average (50 replicates) execution time (standard deviation) of simulating 5-Mb sequence with recombination hotspots (hh:mm:ss)*

**Number of hotspots**	***N***	**ms**	**msHOT**	**MacsCS**	Simcoal2^*^	**Fastsimcoal**
0	100	1:48 (0:03)	1:49 (0:03)	0:17 (0:02)	1:08:23 (4:22)	0:03 (4×10^-3^)
	500	---	---	0:48 (0:03)	1:17:02 (4:41)	0:16 (0:02)
	1,000	---	---	1:39 (0:07)	1:20:57 (7:23)	0:40 (0:02)
	10,000	---	---	29:45 (1:22)	1:55:01 (8:21)	12:36 (0:14)
2	100	---	1:36:18 (8:41)	0:17 (0:03)	1:16:16 (4:30)	0:03 (4×10^-3^)
	500	---	1:54:09 (6:20)	0:51 (0:08)	1:17:29 (6:23)	0:17 (0:03)
	1,000	---	1:59:32 (7:30)	1:32 (0:10)	1:25:50 (7:31)	0:40 (0:03)
	10,000	---	2:02:28 (10:52)	30:02 (3:04)	2:08:31 (7:24)	13:01 (0:10)
5	100	---	3:08:45 (13:11)	0:19 (0:3)	1:09:38 (7:10)	0:04 (0:01)
	500	---	3:23:29 (16:08)	0:49 (0:07)	1:10:59 (10:03)	0:17 (0:02)
	1,000	---	3:31:39 (17:14)	1:34 (0:12)	1:24:10 (9:37)	0:41 (0:05)
	10,000	---	4:12:07 (20:06)	36:50 (5:47)	1:57:03 (14:20)	13:17 (0:12)

* “*N*” denotes sample size (i.e., the number of DNA sequences) for each replicate. “---” denotes a segmentation fault (e.g., ms could not simulate sequence data with recombination hotspots).

### Validation of recombination hotspots

**Figure 4:**
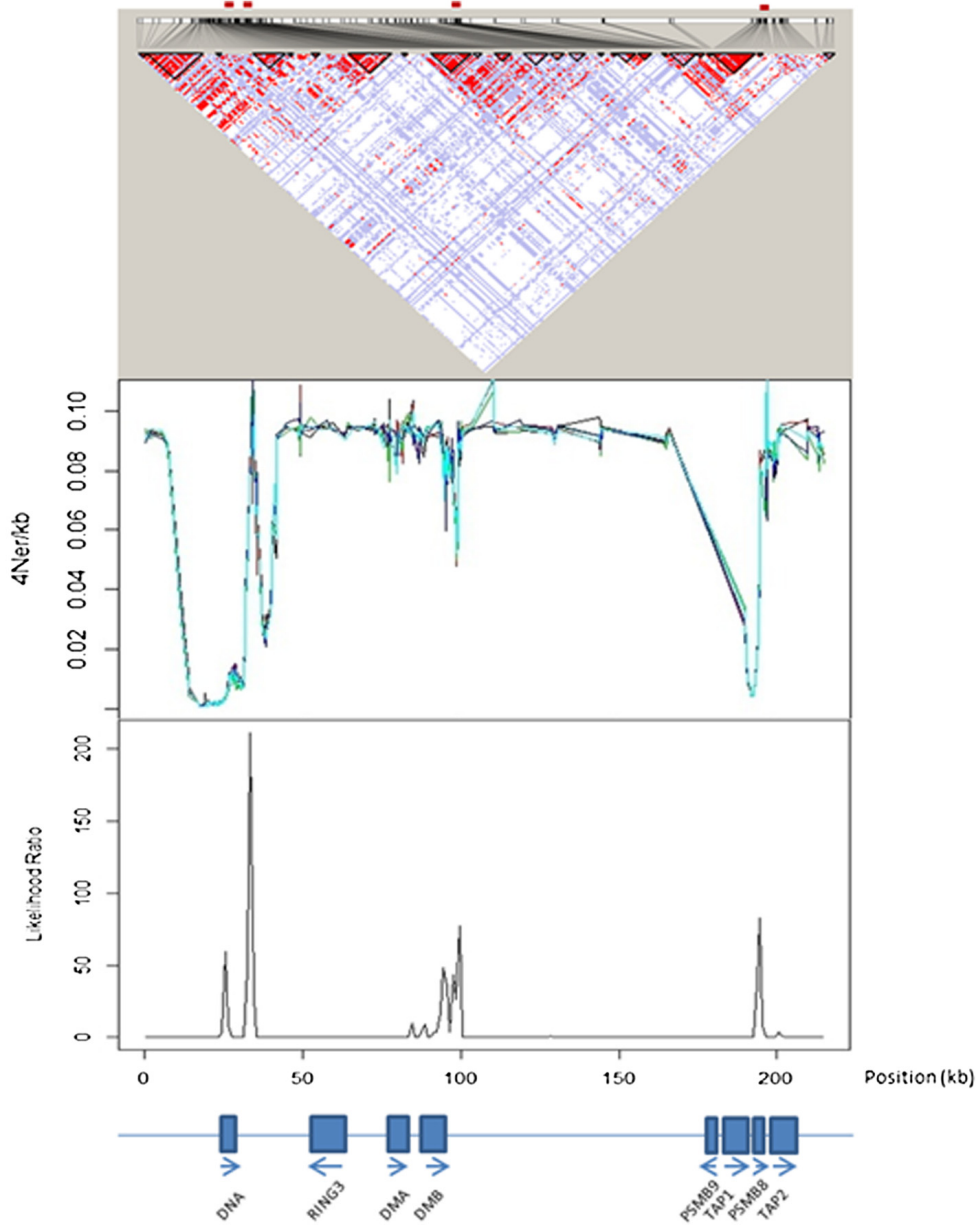
The linkage disequilibrium block structure generated by Haploview for the 216-kb human HLA class II region (total 263 SNPs) based on 100 haplotypes reconstructed by PHASE v2.1 (top panel), LDhat 2.2 rhomap estimation results of five runs for detecting recombination hotspots (middle panel; five different colors denote these different runs), and cross-over hotspot peaks revealed by the likelihood ratio graph generated by sequenceLDhot (bottom panel).

**Figure 5:**
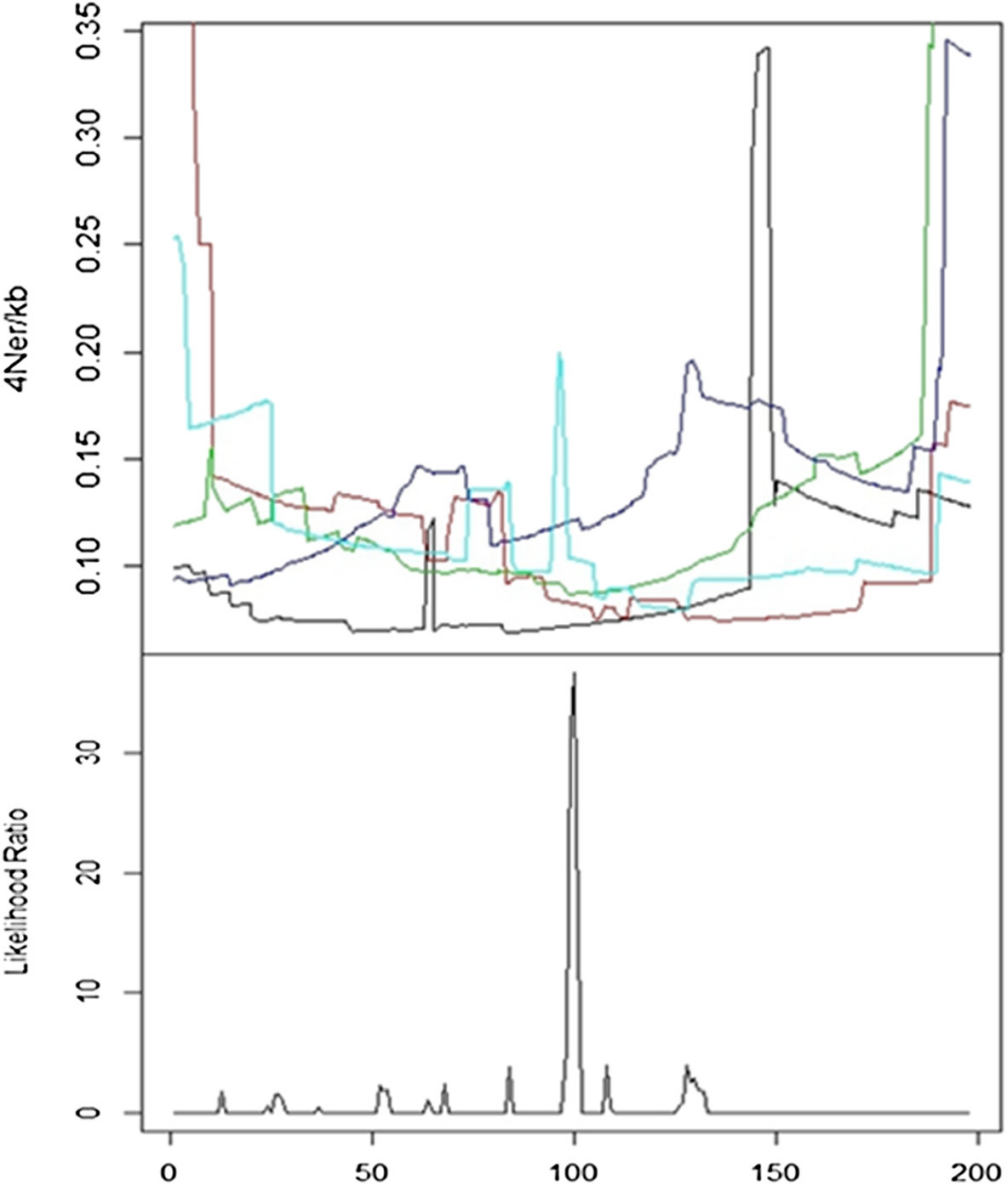
LDhat 2.2 rhomap estimation results of recombination rates for simulation data of a 200-kb DNA sequence for five runs for detecting recombination hotspots in a single simulation data set (top panel; five different colors denote these different runs) (368 SNPs; a total of 100 DNA sequences was simulated based on a 1-hotspot model by MaCS) versus cross-over hotspot peaks revealed by the likelihood ratio graph generated by sequenceLDhot (bottom panel).

**Figure 6:**
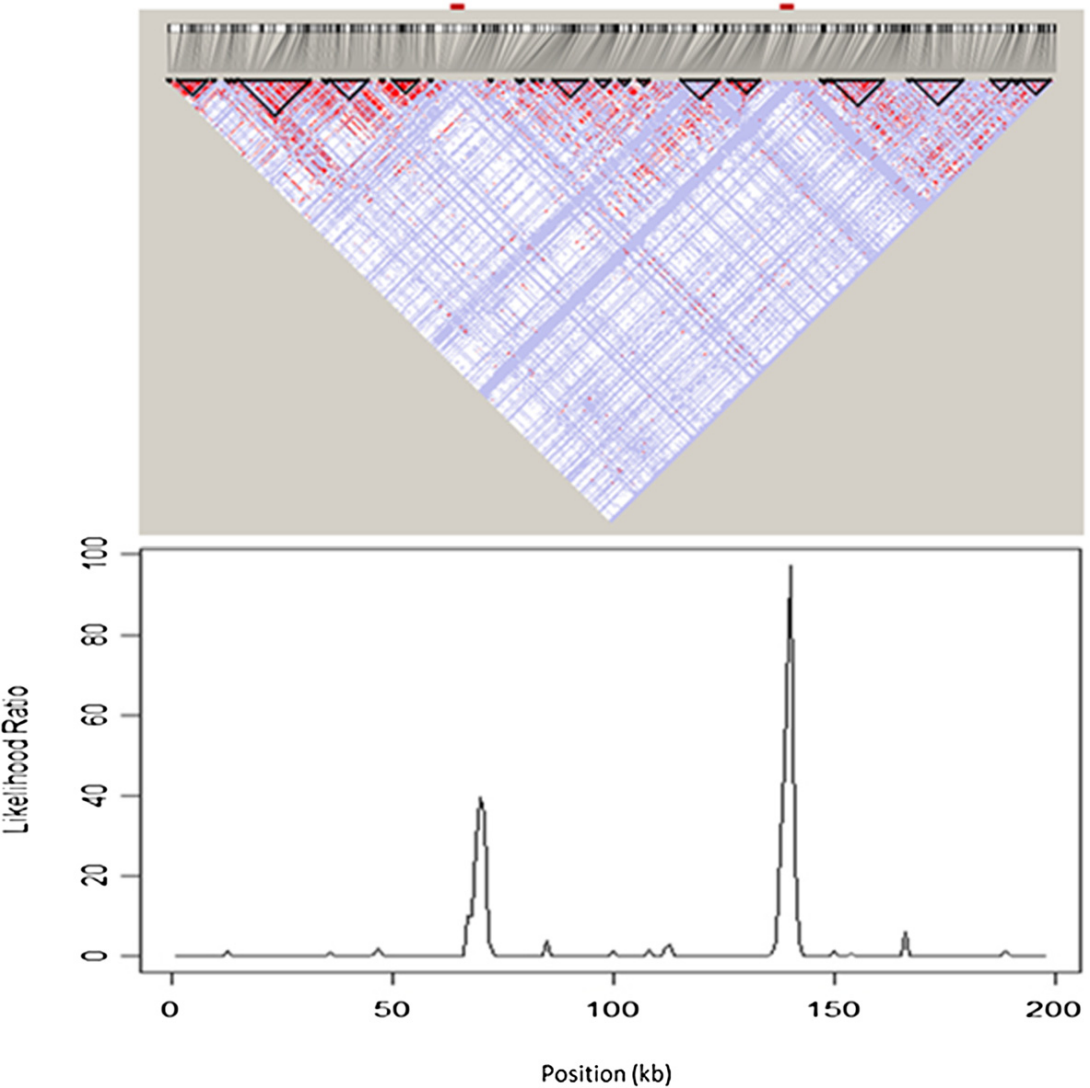
The linkage disequilibrium block structure generated by Haploview for a 200-kb DNA sequence with 2 hotspots (total 459 SNPs) based on 100 DNA sequences simulated under a 2-hotspot model by fastsimcoal (top panel) versus cross-over hotspot peaks revealed by the likelihood ratio graph generated by sequenceLDhot (bottom panel).

For real data based on the HLA class II region, LDhat 2.2 rhomap, sequenceLDhot and Haploview were applied to detect the six recombination hotspots (i.e., DNA1, DNA2, DNA3, DMB1, DMB2, and TAP2) identified by sperm typing [43]. This 216-kb region has a comparable length to that of our simulated data (200-kb). Results of these three hotspot detection programs are shown in Figure 4. The Haploview plot displayed a rugged “block-like” pattern of haplotype structure (top panel), which did not reveal precise locations of these recombination hotspots. Since LDhat 2.2 rhomap adopted the MCMC approach, the estimation of recombination rates along the DNA sequence was performed in five runs in order to determine the consistency across different runs. For each time, we ran rhomap for a total of 10,000,000 iterations including a burn-in of 1,000,000 iterations and taking a sample every 5,000 iterations, As suggested by the LDhat 2.2 user manual, both block penalty and hotspot penalty were set to be 0’s. As shown in Figure 4 (middle panel), moderate variations across these five runs were observed (results shown by different colors), and the positions of the six recombination hotspots were not indubitably discernable. The LR graph for the estimation results by sequenceLDhot provided the most accurate results, correctly identifying five of the six recombination hotspots, but not the leftmost hotspot (i.e., DNA1) which is the weakest hotspot spaced very close to the adjacent DNA2 hotspot (Figure 4; bottom panel). For simulated data, we first simulated a total of 100 DNA sequences (200-kb in length) for 368 SNPs based on a 1-hotspot model by MaCS. LDhat 2.2 rhomap was run five times (results shown by different colors) to detect this recombination hotspot for a single simulation data set. Then, sequenceLDhot was run to detect the recombination hotspot. Among the five runs of LDhat 2.2 rhomap, only one run appeared to identify the correct hotspot peak (100-kb position), and the results across these five runs were not consistent (Figure 5; top panel). By contrast, sequenceLDhot precisely detected the recombination hotspot at the correct position (Figure 5; bottom panel). We also simulated a total of 100 DNA sequences (200-kb in length) for 459 SNPs based on a 2-hotspot model by fastsimcoal. Haploview results gave rise to a rumpled LD pattern that could be barely perceived as three “fuzzy” LD blocks (i.e., left, middle and right), but the precise locations of the two recombination hotspots were far from clear (Figure 6; top panel). However, sequenceLDhot correctly detected the two simulated recombination hotspots at expected 70- and 140-kb positions (Figure 6; bottom panel) unambiguously. Taken together, our results based on both real and simulated data indicate that for hotspot detection, LDhat 2.2 rhomap performed much less accurate than sequenceLDhot, and the visual discernment of recombination hotspots based on the Haploview plot was much less obvious as compared with results of sequenceLDhot. Therefore, sequenceLDhot was chosen as the software tool to validate the recombination hotspots from simulated data by various coalescent simulators, which was shown to be much more robust and accurate in detecting recombination hotspots than the other two computer programs. Twenty replicates for each simulation setting (see Simulation data sets subsection in Methods section) were simulated for each coalescent simulator. Two types of metrics were applied in assessing the accuracy of the simulated recombination hotspots. The first metric is the proportion of simulated hotspots that could not be detected by sequenceLDhot based on a hotspot intensity threshold. The other metric is the extent of shifting of the detected hotspot position from the expected position. We define a recombination hotspot shifting > 1-kb (to either left or right) as a significant shifting. For each recombination, hotspot, sequenceLDhot provided an estimated value of the LR statistic. If the LR exceeded a predefined threshold (denoted as *c*), the hotspot would be treated as a significant one. The higher the LR, the more certain the corresponding hotspot. We chose the default parameter settings such that the width (*w*) of the hotspot was 2,000-bp, and the spacing (*l*) was 1,000-bp. The value of *c* of LR for claiming a significant hotspot was set to be 10. Of the five programs, only msHOT, MaCS, and fastsimcoal were selected for comparisons because ms could not handle a user-specified hotspot model (Tables 1, 2, 3) and Simcoal2 was not so scalable (Table 4).

**Table 4:** Validation results by sequenceLDhot for 2- and 5-hotspot models (20 replicates each) (genomic sequence length = 0.2-Mb)

**Number of hotspots**	**Simulator**	**# Detected peaks/Total # simulated peaks**	**Mean (standard deviation) of LR**	Mean shifting (kb)^*^	**# Significant shiftings**^**†**^**/# Detected peaks**
2	msHOT	39/40	45.83 (18.35)	-26	2/39
	MaCS	38/40	28.73 (23.36)	86.65	4/38
	fastsimcoal	36/40	41.16 (20.75)	10.8	2/36
5	msHOT	91/100	47.49 (28.87)	-981.33	13/91
	MaCS	93/100	43.32 (25.48)	-57	11/93
	fastsimcoal	94/100	48.78 (22.06)	-35	14/94

*A negative number indicates a shifting to the left of the expected position.

^†^A significant shifting was defined as a shifting out of the range defined by ± 1-kb away from the expected peak position.

**Figure 7:**
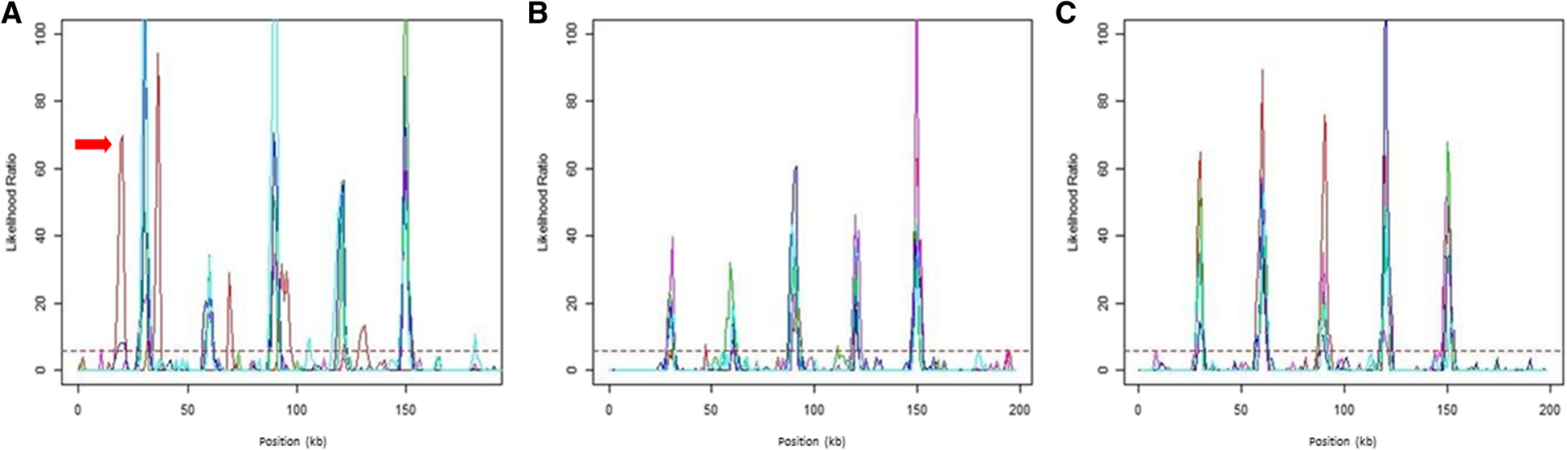
**Validation results of positions and intensities for recombination hotspots (genomic sequence length = 0.2-Mb) simulated under a 5-hotspot model by sequenceLDhot for five randomly selected replicates out of total 20 simulated data sets by (A) msHOT (a red arrow indicates a shifted replicate), (B) MaCS, and (C) fastsimcoal.** In each panel, five different colors denote five different replicates.

When sequence data were simulated according to the 2-hotspot model, sequenceLDhot detected 39 of the total 40 hotspots from data simulated by msHOT, and of the detected ones, two shifted significantly away from their expected positions. The mean shifting of all detected hotspots was 26-kb to the left. It had the highest mean LR (45.83) and the lowest standard deviation (18.35) (Table 4). Data simulated by MaCS had the lowest mean LR (28.73) and the highest standard deviation (23.36), with 38 of the total 40 hotspots detected and 4 of the detected ones significantly shifted. The mean shifting of recombination hotspots simulated by MaCS was 86.65-kb. Hotspots simulated by fastsimcoal had the smallest shifting — a mean of 10.8-kb, but had the least number of significant ones found by sequenceLDhot — 36 of 40. Two of the detected ones were found significantly shifted. Thus, our simulation results based on 2-hotspot model showed that, MaCS had the poorest performance, with the largest mean shifting and number of significant shiftings, whereas msHOT and fastsimocal had comparable performances. For simulations according to the 5-hotspot model, fastsimcoal-simulated data had the best performance, with 94 out of 100 hotspots detected by sequenceLDhot, as compared to 91 out of 100 for msHOT-simulated data, and 93 out of 100 for MaCS-simulated data. Besides, it had the highest mean LR (48.78) and the lowest standard deviation (22.06). Its mean shifting was also the lowest (i.e., 35-kb to the left), while the number of significantly shifted hotspots was comparable to that of MaCS and smaller than that of msHOT. The mean shifting for msHOT was substantially higher than those of MaCS and fastsimcoal, which was 981.23-bp to the left. In our simulations based on the 5-hotspot model, fastsimcoal had the most stable performance and generated the most accurate recombination hotspots whereas msHOT had comparatively the worst performance of the three coalescent simulators. Figure 7 showed positions of hotspot peaks detected by sequenceLDhot from data simulated by msHOT (Figure 7), MaCS (Figure 7B), and fastsimcoal (Figure 7C) according to the 5-hotspot model. For each model, for the sake of clarity, we randomly selected five of the 20 replicates, and then superimposed them on the same graph. Each color represents a replicate. All these five lines were supposedly to be matched perfectly with 5 hotspot peaks at positions 3-, 6-, 9-, 12-, and 15-kb, respectively, if all the simulations were accurate. However, the positions of hotspot peaks detected from data simulated by msHOT were not consistent across the five replicates (Figure 7A). A significant shifting was observed for one of the five replicates (red line), and the LRs of the five hotspot peaks across the five replicates exhibited dramatic variations (Table 4 and Figure 7A). If an LR of 6 was applied as the threshold to define hotspot peaks, 6 (16.13%) were “noisy” (i.e., incorrect) peaks out of the 31 total detected peaks (i.e., LR > 6) (Figure 7A) from data simulated by msHOT. Hotspot peaks detected from MaCS-simulated data appeared more consistent (Figure 7B) — No significant shiftings were detected and 5 (16.67%) “noisy” peaks out of 30 total detected peaks were found. For fastsimcoal-simulated data sets, no “noisy” peaks were found (i.e., all 25 detected peaks were correct) and the positions of these hotspot peaks were the most consistent (Figure 7C). A similar pattern was shown when the LR graph was generated for all 20 data sets simulated by each of msHOT, MaCS, and fastsimcoal (data not shown). Therefore, although msHOT could be applied to simulate recombination hotspot peaks, their positions appear to be less precise compared to those of MaCS and fastsimcoal, at least based on a 5-hotspot model.

## Discussion

Advances in next-generation sequencing technologies have resulted in a dramatic increase in generating whole genome sequence data. There is an urgent need to develop novel methods for analyzing such huge amounts of data. Along with it, computer simulation of genome-wide data is also crucial. Coalescent model has been the most attractive model in population genetics, and is widely recognized as the cornerstone in statistical analysis of DNA sequences [44]. The quintessential feature of coalescent is to start with the current sample of DNA sequences and then trace backward in time to identify past events since their MRCA [44,45]. The standard coalescent provides an accurate characterization of genealogies of haploid individuals of constant size, which can incorporate recombination [13,45,46]. However, the original standard coalescent does have several restrictive features based on the neutral theory that limits its application to real-world DNA sequences, and has been extended to handle selection [47-49], gene conversion [14,50,51], and migration [52-55]. As indicated by [53], the generating models based on coalescent theory should resemble real data as much as possible. Therefore, even the exact standard coalescent might not be the “best” model that could generate simulated data “most” similar to present-day DNA sequences. Nevertheless, the coalescent model based on the Wright-Fisher model is a theoretically convenient and reasonable approximation to real-world scenarios. By incorporating prior information based on coalescent theory, PHASE v2.1 [33,39,40] significantly improved phasing accuracies for both real and simulated data sets. Therefore, standard coalescent remains a widely applied tool in modeling real-world DNA sequences. However, standard coalescent implemented based on full ARGs incurs a high computational cost for a relatively long DNA sequence (e.g., > 5-Mb), making it difficult to simulate DNA sequences at the genome scale for a large sample size (e.g., > 500). To overcome this obstacle, SMC, an approximation to standard coalescent, has been developed which scales linearly with the length of the DNA sequence being simulated from the left to the right, and has the remarkable advantage of being much faster and more extensible than standard coalescent algorithm [15]. Based on variants of SMC, both MaCS and fastsimcoal could generate LD patterns of DNA sequences very close to those generated under a classical ARG model but much more swiftly [17].

After our extensive simulations based on five widely used programs, for simulating up to a few hundred of samples (sequences) with sequence lengths spanning several Mbs, Hudson’s ms is a great choice for its flexibility in handling historical events and robust modeling. For simulating sequences up to tens of or a few hundred of Mbs or a large number of samples, ms is no longer adaptable. In our simulations, ms could not handle 10,000 samples for a 1-Mb sequence or 500 samples for a 5-Mb sequence. The basic algorithm of msHOT [14] is an extended version of ms (which generates ARGs for a sample of chromosomes based on coalescent theory) by adding both recombination hotspot and gene conversion hotspot models to the implementation of standard coalescent by ms. In simulating the simplest scenario of 0-hotspot model assuming also no gene conversions, the implementation of msHOT appears to be the same as that of ms (because there is no necessity to invoke complex cross-over and gene conversion hotspot models). However, when there is at least one recombination hotspot in the simulated DNA sequences, the implementation of msHOT algorithm must differ from that of ms to account for the presence of recombination hotspot(s) in coalescent simulation. As stated in [14], the modification of msHOT allows the user to insert any pre-specified non-overlapping cross-over hotspots and non-overlapping gene conversion hotspots into the genetic region by specifying the locations and intensities for each. Specifically, incorporating *R* recombination hotspots requires the user to specify a left endpoint (*a*_*h*_), right endpoint (*b*_*h*_), and intensity (*I*_*h*_) for each hotspot *h*, where *h* = 1, …, *R*. Inside a given hotspot *h*, the probability of a recombination occurring between two adjacent base pairs in a single transmission from parent to offspring is *λ*_*h*_*r*_*bp*_. Outside recombination hotspot(s), this probability is the recombination probability per base pair — *r*_*bp*_. That is the reason when for simulating scenarios for 2- and 5-hotspot models, msHOT performance appears to be much slower (to account for the extra complexity introduced by the user-defined recombination hotspots) than for the 0-hotspot model. In addition, in the absence of a recombination hotspot, just like ms, msHOT could not handle a sample size of 10,000 sequences for simulating 1-Mb DNA sequence data (Table 2) or sample sizes of 500, 1,000, and 10,000 for simulating 5-Mb DNA sequence data (Table 2). However, in the presence of at least one recombination hotspot, by taking a very different implementation compared to ms by including a more complex hotspot model, msHOT could handle such large sample sizes. By contrast, MaCS, which is based on a modified SMC algorithm, could achieve coalescent simulations for many more samples with much longer length, while accurately approximating the results simulated by standard coalescent (i.e., ms) and maintaining its flexibility. Theoretical interpretations for empirical observations of Table 3 are as follows. When simulating 2- and 5-hotspot models for relatively long DNA sequences (i.e., 5-Mb), msHOT (built on an extended algorithm based on ms) and Simcoal2 [built on a discrete generation-by-generation approach (rather than a continuous time approximation)] are understandably much slower than MaCS and fastsimcoal due to their critical algorithmic differences — MaCS has taken a faster modified SMC approach and fastsimcoal has also taken a computationally more efficient SMC’ approach. As indicated in Background section, the SMC method of [22], and the SMC’ method of [10] are both approximations to the standard coalescent algorithm, which have the advantage of being much faster. Specifically, MaCS [15] is a generalized SMC which is equivalent to SMC when the “history” parameter *h* = 1 bp, but becomes a closer approximation to ms than SMC when *h* increases (such that more information of adjacent genealogies are stored). Generally speaking, MaCS produces simulated data that are virtually identical to data simulated under the standard coalescent, but in much less time and using much less memory [15]. Similar to MaCS, fastsimcoal is based on a continuous-time SMC’ by applying ABC, which is much faster than msHOT or discrete generation coalescent approach of Simcoal2 that also gives excellent approximations to standard coalescent with a much quicker speed [17].

Based on cross-over hotspot validation results (Table 4 and Figure 7), recombination hotspots simulated by MaCS did not appear to be as accurate as those of msHOT and fastsimcoal for a 2-hotspot model. For a 5-hotspot model, MaCS outperformed msHOT, but fastsimcoal was the most accurate simulator. When there is a demand for simulating DNA sequences under a variety of population genetic models [especially in the presence of cross-over hotspot(s)], fastsimcoal appears to be the best choice. Different data types [DNA, SNP, simple tandem repeat (STR)] and sequences of different structures could be simulated by fastsimcoal, in addition to its advantages in terms of efficiency, accuracy, and capability of generating any user-defined patterns of recombination hotspots. From a practical standpoint, when a set of recombination hotspots need to be simulated, msHOT, MaCS, and fastsimcoal are all applicable, but msHOT performed much slower than MaCS and fastsimcoal, and had the lowest accuracy based on validation results of sequenceLDhot.

## Conclusions

While Hudson’s ms remains an excellent choice for simulating relatively short DNA sequences (< several Mbs) under general scenarios, MaCS and fastsimcoal are much more scalable and flexible in simulating many different demographic histories and diverse DNA sequence structures (e.g., SNPs and STRs). Based on both running time and hotspot validation comparisons, fastsimcoal is shown to be the fastest and most reliable and consistent coalescent simulator, especially when the number of hotspots is large. MaCS is a runner-up with a lower speed and a slightly less accuracy. Based on our extensive simulation evaluation and comparison results, cautions should be taken in applications of these widely used coalescent simulators, such that sequence data simulated by a given software should be checked and validated — e.g., the positions and intensities of recombination hotspots, to guard against any discrepancy between the intended objective and the actual simulation results. Further, for detecting and validating recombination hotspots, among the three widely used computer programs, sequenceLDhot appears to be the best choice— fast, robust and accurate. In real-world DNA sequence data, a variety of factors such as GC content, local LD block structure, DNA elements that act as enhancers or inhibitors of recombination [56-58], could affect the intensities and locations of recombination hotspots along a given chromosome. For example, recombination hotspots correlate positively with GC content [59,60]. Further, certain DNA motifs are enriched in cross-over hotspots, among which CCTCCCT and CCCCACCCC are the most prominent [61]. Thereafter, in real-world scenarios, these factors should be taken into consideration in identifying genuine cross-over hotspots.

## Abbreviations

ABC: Approximate Bayesian Computation; ARG: Ancestral recombination graph; LD: Linkage disequilibrium; LR: Likelihood ratio; HLA: Human leukocyte antige; MCMC: Markov Chain Monte Carlo; MRCA: Most recent common ancestor; SMC: Sequential Markov coalescent; SNP: Single nucleotide polymorphism; STR: Simple tandem repeat.

## Competing interests

The authors declare that they have no competing interests.

## Authors’ contributions

TN conceived the idea for the project, TN and HWD contributed to study design and conception, TY and TN participated in data simulation, analysis and interpretation, TY and TN drafted the manuscript and HWD revised it critically for intellectual content. All authors read and approved the final version of the manuscript.
